# Medical Opinion and Movement

**Published:** 1909-09-11

**Authors:** 


### Medical Opinion and Movement.

At a recent meeting of the Chicago Gynæcological Society Dr. L. E. Frankenthal described a method of obtaining the advantages of Walcher's position without the necessity for one or more special nurses, without discomfort to the patient, and without horrifying any relatives that may be present. He has a harness, constructed of towelling which consists of three parts. By one bandage, which is applied first, the pelvis and abdomen are fixed to the edge of the bed towards which in the lateral position the patient is facing. This broad bandage passes round the lumbar and sacral regions and fixes the pelvis in such a way that the other parts of the harness may produce full extension of the vertebræ. The next applied is a broad band, made with holes for the arms, which bears chiefly on the front of the chest, and is fixed to the opposite side of the bed to the pelvis band. It thus draws backwards the shoulders and extends the dorsal spine. Lastly, the ankles are tied together and to the same side of the bed as the shoulder bandage, with the thighs hyper-extended and the knees flexed. After a little time the extension of the thighs can be increased by gently pushing the knees backwards, at the same time tightening the ankle bandage. Patients do not beg to be released from this contrivance, though three hours is the longest for which continuous application has been ordered. Bodily warmth can be better maintained than in Walcher's position, and there is no œdema of the feet or soft parts. The hyperflexion of the anterior part of the pelvic inlet upon the sacroclias joints is obtained just as effectively as by the Walcher plan, for the approach of the lower sacrum and coccyx to the ilium, and the consequent relaxation of the soft parts, are equally well effected.

A most useful form of Treatment of Chronic Constipation, which perhaps is of more frequent application in Germany than elsewhere, is the methodical employment of oil enemata. There are, however, certain objectionable features attached to this procedure, among which may be mentioned the soiling of linen, the necessity of lying down after the operation, the escape of foul-smelling gases due to decomposition of the oil, and in some cases abdominal pain, malaise, and intestinal irritation. Lipowski, in the *Berliner Klinische Wochenschrifte*, proposes to overcome these difficulties while at the same time retaining and even increasing the good effects of the oil enema, by substituting for it a mixture of one part of solid with eight of liquid paraffin. Having previously warmed it to a temperature of 100° Fahr., the patient seated at the edge of a chair introduces about seven ounces of the mixture into his rectum by means of a syringe and soft catheter. The paraffin has no irritant action on the intestinal mucous membrane, does not decompose, and has a greater purgative effect than oil. Since it is quickly deposited on the mucous membrane of the bowel in the form of a pomade, and has no tendency to escape from the anus, there is no necessity for the patient to lie down after taking the enema. The author has shown experimentally that resorption of water from the large intestine is much increased in cases of chronic constipation. Oil has the faculty of delaying this resorptive process, and paraffin is even more effective than oil.

Accidents of Electrical Origin to the Eye are comparatively rare and usually not very severe, but there are three cases on record in which the accidental passage of a strong electric current through the body resulted in the development later of a cataract. A fourth case of this kind has just been published by Terrien. A current of 550 volts passed through the body of a young electrician while he was repairing a machine. Picked up unconscious, he was removed to hospital, where he regained his senses in a couple of hours. His injuries comprised moderately extensive facial burns with some slight conjunctival injection; there was, however, but slight reaction, and both eyes and skin rapidly returned to their normal condition. It was not until three months after the accident that further complications set in, resulting in the formation of a cataract limited at first to the subcortical layers. In the course of six weeks, however, complete blindness ensued from the involvement of the remaining layers of the lens. From the study of this and the three previous cases, the author concludes that cataract appears comparatively late after the accident—*i.e.* in from six to twelve weeks, and that it may be complete and lead to total blindness in the affected organ; prognosis in this class of accident must therefore be very guarded. The pathology of the condition is obscure, but perhaps the most likely explanation is that the current has mechanical and electrolytic action on the crystallin of the lens. Whatever be the pathological explanation, the injury entails a disturbance of the lens with relaxation of the ligaments which renders subsequent extraction difficult, and the prognosis still more unfavourable.

The term Ozœna is often loosely employed to denote any condition of the nasal passages in which fœtor is the predominant symptom. Lavrand, in the *Journal des Sciences Médicales de Lille*, draws attention to the impropriety of this, and gives a definition of the only condition to which the term should be applied. Ozœna is a morbid entity with a very exact symptomatology—namely, a fœtor peculiar to itself, the presence in the nasal passages of dried secretion in the form of adherent grey or green crusts, and lastly total atrophy of the mucous membrane and walls of the cavities. It is thus easily distinguishable from such affections as syphilitic necrosis, suppurative sinusitis, and rhinitis following the presence of foreign bodies, all of which are accompanied by more or less fœtor. As the result of repeated examination of a large number of cases, the author came to the conclusion that the crusts are the result of a secretion coming from the middle meatus, and on probing this passage he was able to show that, in all cases of true ozœna, a condition of ethmoidal osteitis is present, varying in extent according to the severity of the case, and uni- or bi-lateral according as the ozœna is present on one or both sides. In no case was he able to find disease of any other sinus than the middle one, so that the ethmoid alone is affected. Treatment therefore is purely surgical, and the results of curetting have been most satisfactory in his hands. The old treatment by paraffin, which was supposed to reduce the size of the cavities and so restrict the secretion, must therefore be abandoned, for it is obvious that the increase in size of the sinuses is the result, not the cause, of the disease. The atrophy can be explained by the osteitis producing a neuritis of the trophic nerves in its neighbourhood.

---

While the effect of the X-rays upon the Cancer Cells of superficial growths is now beyond dispute, it has to be admitted that the penetrating power of the rays for deeper growths does not appear to be very great. Information is scanty as to how deep the rays penetrate and influence the cancer cells, but some light has been thrown on the question by the work of M. E. Bornait-Legueule. He has examined histologically ten cancers of the breast that have been treated previously with the Röntgen rays. He finds that the most marked effect of the rays upon the cancer cells was shown in the superficial layers. To a depth of 10 or 12 millimetres from the skin the neoplastic elements had disappeared, and they were more or less modified though not destroyed, as far as 25 mm. But at a depth of 3 c.m. living cancer cells were found in the midst of others undergoing involution, and this in spite of large doses of the rays. The neoplastic cells appear to be eliminated by means of an intensive phagocytosis, by which the growth becomes invaded and the epitheliomatous lobules encircled. Charged with the cellular débris of the neoplasm the phagocytes disappear and are replaced by a proliferation of connective tissue elements,' already demonstrated by Bashford and by Exner. When this development of connective tissue is not much in evidence the disappearance of the neoplastic cells leaves lacunar spaces around the cancer lobules that have resisted the action of the rays. These lacunæ or fissures are of especial interest showing that the $x$-rays have an elective action upon the cancer cells apart from any vascular changes or connective tissue proliferation.

---

According to the observations of certain authorities, particularly Vyssokovitch, Bacteria circulating in the Blood cannot penetrate healthy kidneys, and so find their way into the Urine. The recent researches, however, of Vincenzi do not confirm this supposition. He worked with a bacillus coli virulent for guinea pigs and rabbits, and he found that the healthy kidneys in these animals were quite permeable to this microbe. He injected 1 to 2 c.c. of bouillon culture of the bacillus coli into the jugular vein, and after two to five hours killed the animals by chloroform. Having ligatured the bladder close to the urethra, he drew it out from the abdominal cavity, and with aseptic precautions aspirated it. Agar and gelatine cultures were then made from the urine drawn off. Without exception all the cultures gave positive results. The urines showed otherwise no abnormality, neither albumen, epithelial cells nor red corpuscles, and the kidneys examined microscopically were found to be quite healthy. A careful examination of the renal sections showed in some cases a few bacilli in the glomeruli, and some were also detected in Bowman's capsules. It appears therefore from these researches that at least some bacilli are able to pass through the walls of the capillary blood-vessels, without any accompanying appreciable changes in the vessel walls, especially in parts such as the glomeruli of the kidneys where there is a slowing of the blood-stream, and in this way they may pass from the general circulation into the urine.

---

By Radioscopic Examination after the administration of bismuth subnitrate, Dr. K. Faber has made some interesting observations on the position of the stomach in the normal subject and in cases of gastroptosis. By examining a number of young subjects of both sexes in good health he finds that in males the stomach has the form of a vertical bag or sack, the lower end of which is bent upwards. It lies a little to the left of the middle line and the lower end crosses this line to end in the lower right half of the epigastrium. The greater curvature passes almost vertically down to 1 to 3 c.m. above the umbilicus, where it crosses the middle line, but the extent of this vertical portion forms the chief variation in different subjects. In tall thin males it may extend to the umbilicus or even below, and in females it is much lower than in males. Among 70 female subjects examined, in only two or three cases did the greater curvature lie altogether above the umbilicus. In cases of gastroptosis the chief characteristic consists in the length of this descending vertical portion of the stomach which may measure as much as 30 cm., whereas normally it should not exceed about 25 cm. Dr. Faber considers that in women a stomach is to be regarded as abnormal when the lesser curvature reaches to the level of, or below the umbilicus. In man a ptosis of that extent is very rare. On the other hand he finds that clinically a very considerable amount of gastroptosis may be present, especially in women, without giving rise to symptoms. He thinks that when symptoms do arise they are due to an accompanying gastritis, or to a loss in neuro-muscular tone. In such cases the condition is naturally aggravated by the ptosis of the organ causing increased difficulty in the evacuation of the stomach contents.

---

Adopting the hypothesis that the œdema of Bright's disease is due to some toxin, Dr. Timofeew has carried out some interesting experiments on dogs to determine the presence and origin of the toxin. An account of the work appears in the *Archives für Experimental Pathologie und Pharmacologie*. Changes in the lymph flow were observed by establishing a fistula in the thoracic duct. Intravenous injection of normal serum showed no increase in the lymph flow, and the same result obtained after double nephrectomy. From this the author argued that œdema is not produced by toxic bodies retained from failure of excretion by the kidneys, but that the supposed toxin is probably in the renal cells themselves. Subsequent experiments appear to confirm this. Ligature of a ureter or renal artery of one side produced considerable increase in the lymph flow, and the serum of dogs so treated injected into other dogs proved highly lymphagogic. The coagulation of the blood and lymph was found also to be retarded. Moreover normal saline injected intravenously into dogs after ligature of the renal artery produced effusions in the serous cavities, pulmonary œdema, and sometimes subcutaneous œdema, whereas no such effects were observed in normal dogs. Dr. Timofeew concludes from these researches that the kidneys themselves form these toxic bodies, which he proposes to term "nephroblastins," and which, in cases of inflammatory changes of the kidney cells, pass into the general circulation and produce the œdemas characteristic of certain forms of renal disease.

---

A METHOD of Treating Abscesses by Repeated Puncture has been described recently by Ritter in the *Berliner Klinische Wochenschrifte.* A trocar and cannula of diameter sufficiently large to ensure the complete evacuation of the pus are introduced into the abscess. The orifice of the opening is then closed with a collodion dressing. On the following day a similar puncture is made through a second opening, and fluid is drawn off which is usually only slightly purulent. Two days later the operation is repeated, when the fluid will be found to be free from pus. Every second day for a couple of weeks the punctures are continued, after which a complete cure is said to result, even in the case of a large abscess. The author first tried his method on cases of mammary abscess, and owing to the success which attended his efforts extended it later to cases of suppurative adenitis, furunculosis, purulent arthritis, and whitlow. In the last-mentioned disease repeated puncture, if instituted sufficiently early, will ensure the preservation of a phalanx, which at first sight seems beyond hope of salvation. From an æsthetic point of view the results of this treatment will no doubt compare favourably with those obtained by the ordinary method of open operation and drainage, in which an ugly scar is apt to remain after the wound has healed. In cases where æsthetic considerations are of secondary importance the new method cannot however be recommended until further trial has conclusively demonstrated that it is as efficacious as that usually employed. The punctures must be extremely painful, especially with trochars of large calibre, and must necessitate repeated anæsthesia.

---

THE following brief notes on an outbreak of Scarlet Fever seem to be of interest from the point of view of the latency of scarlatinal infection in houses. Dr. W. E. Gladstone, District Health Officer of Dunedin, New Zealand, states that: "In investigating an outbreak of scarlet fever at a cottage in a country settlement a few miles from Dunedin, I found that seven years ago the mother had contracted scarlet fever. After the patient had recovered, the whole building (three small rooms) was fumigated with sulphur, and new wall-paper was pasted over the old wall-paper. During a recent spell of wet weather the roof of the cottage sprung several leaks, the water loosening some of the wall-paper to such an extent that the father thought it desirable to pull all the paper from the walls. The three children played with the paper, and four days later contracted scarlet fever. The children had not been visiting any family, nor had there been anyone visiting the cottage for some weeks."

---

OF the many different methods that have been recommended for the Treatment of Chronic Ulcers of the Skin a comparatively new one devised by Dr. M. J. White, consists in the use of equal parts by weight of thymol iodide and dried ferrous sulphate in powder form. The ulcer is first washed with dilute carbolic lotion, all crusts being removed both from the surface and from the borders. It is then gently but carefully dried, and covered with the powder. As soon as the ferrous sulphate produces a burning sensation the excess of powder is blown off, leaving only a thin adherent film which is now covered thickly with zinc ointment, gauze, strips of adhesive plaster, and a bandage. The dressings should be renewed every other day, and excess of the powder should be avoided.

---

IN the *Gazette des Hôpitaux* attention is drawn by Hallopeau to the advantages of Combining Local with General Antisyphilitic treatment when dealing with cases of Hunterian chancre. The method he advocates is troublesome, both from the point of view of the patient and of the practitioner; but this disadvantage is more than compensated by the possibility of aborting the attack before the onset of secondary symptoms, a result which the author claims to have achieved in a large proportion of his own cases. The chancre is first covered by a pomade containing 30 per cent. crystalline atoxyl, known commercially as diadermine. Round the periphery of the lesion daily injections of 1.5 grs. of atoxyl are given hypodermically. At the same time 15-30 grains of potassium iodide are given each day by the mouth, and intramuscular injections of a mixture containing .3 gr. mercury benzoate, 1 drachm of distilled water, and 5 grs. of glucose or saccharose, are made into the buttock daily. Treatment is continued for six weeks unless the atoxyl gives rise to ocular symptoms, whereupon the local injections are stopped immediately. In some cases, and in all over fifty years of age, Conneyrat's Nectine, a derivation of atoxyl, is substituted for the drug itself. The former is less toxic and more active than atoxyl. The author maintains that these local injections are six times as effective as the general ones in killing the treponema pallidum, and, moreover, prevent the advent of adenitis and roseola. Treatment is well borne by most patients, and is successful in proportion as it is undertaken early.

